# Immune Cell Activation in the Cerebrospinal Fluid of Patients With Parkinson's Disease

**DOI:** 10.3389/fneur.2018.01081

**Published:** 2018-12-18

**Authors:** Jens B. Schröder, Matthias Pawlowski, Gerd Meyer zu Hörste, Catharina C. Gross, Heinz Wiendl, Sven G. Meuth, Tobias Ruck, Tobias Warnecke

**Affiliations:** Department of Neurology, University Hospital Münster, Münster, Germany

**Keywords:** Parkinson's disease, cerebrospinal fluid, immune cells, T lymphocytes, monocytes

## Abstract

**Background:** Parkinson's disease (PD) is a common neurodegenerative disorder. The contribution of the immune system to its pathogenesis remains incompletely understood.

**Methods:** In this study, we performed comprehensive immune cell profiling in the cerebrospinal fluid (CSF) and peripheral blood (PB) of PD patients. Ten PD patients were diagnosed according to brain bank criteria and underwent detailed clinical examination, magnetic resonance imaging, PB and CSF immune cell profiling by multiparameter flow cytometry, and cytokine and chemokine measurements by bead-based arrays. Thirteen healthy elderly volunteers served as control population.

**Results:** The proportions of activated T-lymphocytes and non-classical monocytes in the CSF were increased in patients with PD compared to the control group. In accordance, we found increased levels of the pro-inflammatory cytokines IL-2, IL-6 and TNFα and of the monocyte chemoattractant protein 1 (MCP-1) in the CSF of the included PD patients.

**Conclusions:** Our data provide novel evidence for a response of the innate and adaptive immune system in the central nervous system of patients with PD.

## Introduction

Parkinson's disease (PD) represents the most common neurodegenerative disorder following Alzheimer's disease (1). PD prevalence and incidence increase exponentially with age and peak beyond the age of 80 years (2). Clinically, PD is defined by the presence of classical parkinsonian motor symptoms, accompanied by a variety of non-motor features (3–5).

PD is a slowly progressive neurodegenerative disorder characterized by an early prominent death of dopaminergic neurons in the substantia nigra (6). Affected neurons display cytoplasmic accumulation of proteinaceous aggregates called Lewy bodies, which are mainly composed of α-synuclein and ubiquitin (6). PD results from a complicated interplay of genetic and environmental factors (1). Accumulating research provides evidence for a prominent response of the innate and adaptive immune system in PD (7, 8). In humans, neuropathological studies demonstrated the presence of activated microglia, the resident macrophages of the central nervous system (CNS), in the substantia nigra and other affected brain regions. Additional post-mortem studies found infiltration of the substantia nigra by T lymphocytes, the presence of activated astrocytes, and increased parenchymal cytokine levels (9–13). Functional brain imaging studies detected significant microglial activation in various regions of the CNS (14–16). In particular, PD patients with dementia revealed widespread cortical microglial activation in addition to the subcortical changes (17, 18). Patients with PD have increased cerebrospinal fluid (CSF) levels of inflammatory chemo- and cytokines, including tumor necrosis factor α (TNFα) and interleukin (IL)-6 (12, 19, 20). Genetic studies identified several polymorphisms in genes that are involved in inflammatory processes, such as *TNF, IL1B, CD14*, and *TREM2*, as risk factors for PD (21, 22). Moreover, genome wide association studies revealed *HLA-DRB5* as another susceptibility locus (23).

CSF was shown to reflect many biochemical and cellular events within the brain parenchyma. It is easily accessible in clinical practice and routinely obtained for a variety of diagnostic purposes. Immunophenotyping of CSF cells may be useful to gain insight into the pathophysiology of CNS disorders, and sharpen diagnostic accuracy or estimation of individual prognosis (24).

In this study, we aimed to determine the immune cell profile in the CSF of patients with PD by using multiparameter flow cytometry. We found an intrathecal increase of non-classical monocyte and activated T-lymphocyte proportions in the PD group compared to healthy elderly controls. Correspondingly, we detected increased concentrations of pro-inflammatory cytokines and the chemokine MCP-1 in the CSF of PD patients. Together, these data provide new lines of evidence for a role of both innate and adaptive immune responses in human PD.

## Methods

### Protocol Approval, Registration, and Patient Consent

All patients were recruited from the movement disorder unit at the Department of Neurology, University Hospital Münster, Germany. All participants in this study gave written informed consent. The study was approved by the local ethics committee (2014-624-f-S).

### Participants and Study Population

We included 10 PD patients that presented to our movement disorder clinic. Patients were diagnosed according to the UK Brain Bank criteria. Exclusion criteria for this study were concomitant autoimmune diseases, anti-inflammatory co-medication (e.g., cytotoxic agents, steroids, non-steroid analgesia), evidence of an acute systemic inflammatory process at the time of CSF withdrawal (elevated erythrocyte sedimentation rate above 25 mm/h, C-reactive protein above 0.5 mg/dL, or leukocytes above 11 × 10^3^/μL), or blood-tinged CSF. Patient characteristics are described in detail in Table 1. The CSF control group consisted of 13 healthy elderly participants with a normal neurologic examination. There was no statistical difference regarding their age. CSF was collected during spinal anesthesia before hip replacement and yielded, in all cases, normal cell counts and protein levels.

**Table 1:** Demographics and clinical characteristics of study participants.

**Demographics**	**PD (*n* = 10), CTRL (*n* = 13)**
Age, range [y] (controls)	79, 69–82 (68, 50–79)
Gender [female, %] (controls)	20 (80)
**CLINICAL CHARACTERISTICS**	
PD type [hypokinetic-rigid/mixed, %]	50/50
Disease duration [years]	3 ± 3.96
Hoehn and Yahr stage (25)	3 ± 0.75
L-Dopa/equivalent dose [mg/d] (26)	612.5 ± 324.38
MDS-UPDRS-ON III score (27)	16 ± 6.25
MoCA score (28)	23.5 ± 9.30
**CSF-ANALYSIS**	
CSF protein [mg/l] (controls)	553 ± 271 (368 ± 94)

*Numbers represent the mean ± standard deviation. y, years; disease duration, time between the first reported symptoms and the diagnosis; CTRL, healthy elderly controls*.

### Multiparameter Flow Cytometry and CSF Analysis

Multiparameter flow cytometry of immune cells in PB and CSF samples was done as described previously (24, 29). During lumbar puncture CSF was sampled into polypropylene tubes. All CSF samples were processed in < 20 min. Cells were isolated from CSF by centrifugation (15 min, 290 g, 4°C) and subsequent incubation in VersaLyse buffer (Beckman Coulter, Germany). PB samples were collected in EDTA monovettes and cells were isolated by using VersaLyse buffer. For immunostainings, the following fluorochrome-conjugated antibodies were used: CD14-FITC, CD138-PE, HLA-DR-ECD, CD3-PC5.5, CD56-PC7, CD4-APC, CD19-APC-Alexafluor700, CD16-APC-Alexafluor750, CD8-PacificBlue, and CD45-KromeOrange (all from Beckman-Coulter). Data acquisition was performed with a Navios flow cytometer (Beckman-Coulter). Gating strategy for Leukocytes and Monocytes is described and illustrated in Supplementary Figure 1.

### Quantification of Cytokines and Chemokines in the Serum and Cerebrospinal Fluid

CSF was sampled, and supernatants were obtained by centrifugation as described above. The CSF supernatants were then stored at −20°C until analysis of cytokines (IL-2, IL-4, IL-5, IL-6, IL-9, IL-10, IL-13, IL-17A, IL-17F, IL-21, IL-22, IFNy, TNFa) and chemokines (CCL11, CCL17, CCL20, CXCL1, CXCL5, CXCL9, CXCL11, IL-8, IP-10, MCP-1, MIP-1a, MIP-1b, RANTES) using a bead-based cytokine array (LEGENDplex; BioLegend) according to the manufacturer's instructions.

### Statistics

Statistical analysis was performed using Graphpad Prism 6. All data are reported as mean ± standard deviation, and the pre-chosen significance level for all confirmatory tests was *p* < 0.05. Flow cytometry data were analyzed by using the Mann-Whitney test, presuming a non-Gaussian distribution.

## Results

### The Immune Cell Profile of Patients With PD

**Figure 1:**
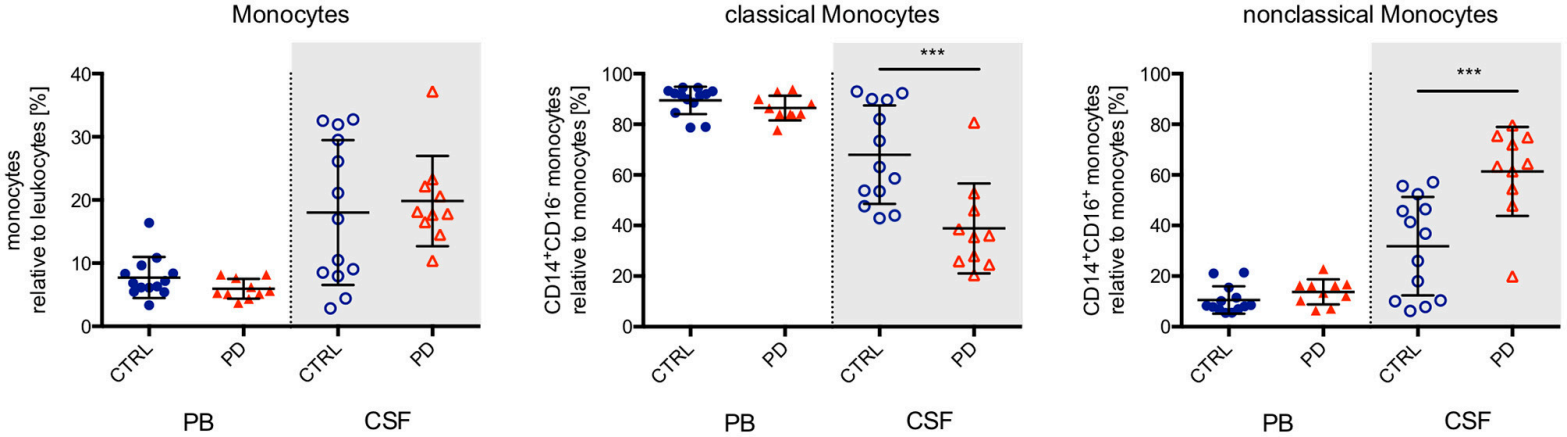
Monocytes in the peripheral blood and cerebrospinal fluid. The proportion of monocytes in relation to total leukocytes was determined in the peripheral blood (PB, closed symbols, white background) and cerebrospinal fluid (CSF, open symbols, gray background) from healthy elderly controls (*n* = 13; blue circles) and PD patients (*n* = 10; red triangles). While the proportion of total monocytes did not differ between study groups, monocyte subgroup analysis based on expression of the surface marker CD16 demonstrated a highly significant decrease of classical monocytes (CD14^+^CD16^−^) and a concomitant highly significant increase of non-classical monocytes (CD14^+^CD16^+^). For statistical analysis, the Mann-Whitney test was performed (***p < 0.001).

**Figure 2:**
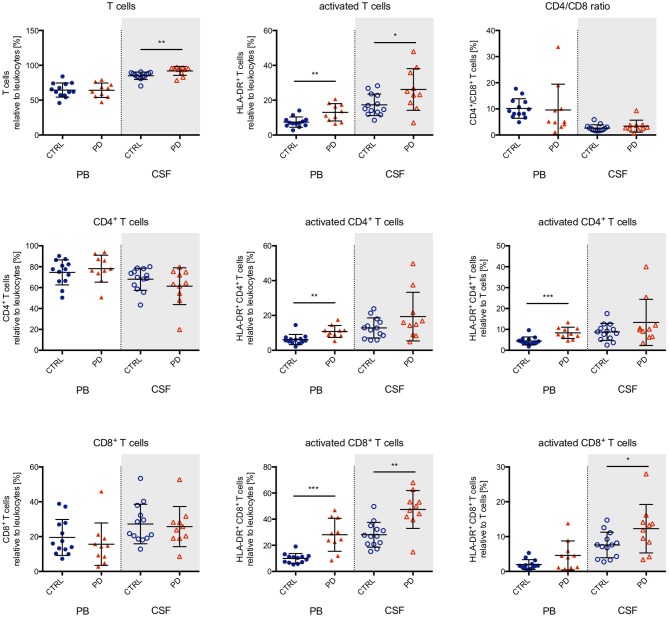
Lymphocytes in the peripheral blood and cerebrospinal fluid. The proportion of T lymphocytes in relation to total leukocytes was determined in the peripheral blood (PB, closed symbols, white background) and cerebrospinal fluid (CSF, open symbols, gray background) from healthy elderly controls (*n* = 13; blue circles) and PD patients (*n* = 10; red triangles). We found a significant increase of total and activated T lymphocytes in the CSF. CD4 and CD8 expression did not differ between study groups, but specifically CD8+ T lymphocytes displayed a larger fraction of HLA-DR expressing activated cells, both in the PB and CSF. For statistical analysis, the Mann-Whitney test was performed (*p < 0.05, **p < 0.01, ***p < 0.001).

Multicolor flow cytometry did not detect significant differences in the proportions or absolute numbers of granulocytes, monocytes, and lymphocytes in the CSF of patients with PD compared to healthy elderly controls (Figure 1, and data not shown). Interestingly, subpopulation analysis of innate immune cells revealed an intrathecal shift in cell proportions from classical monocytes (defined as CD14^+^/CD16^−^) to non-classical monocytes (CD14^+^/CD16^+^; Figure 1). This intrathecal shift was not reflected in the PB (Figure 1). Adaptive immune cell subgroup analysis demonstrated no differences in the levels of B lymphocytes (data not shown), but an increase in the fractions of both total T lymphocytes and activated (defined by HLA-DR expression) T lymphocytes in the CSF (Figure 2). The CD4/CD8 T lymphocyte ratio remained unchanged, but specifically CD8^+^ T lymphocytes showed a larger fraction of HLA-DR activated cells (Figure 2). Both CD4^+^ and CD8^+^ T lymphocyte activation was also increased in the PB (Figure 2). However, we detected no significant alterations in the absolute cell numbers of monocyte or T lymphocyte subsets (data not shown).

### The Cytokine and Chemokine Profile of Patients With PD

**Figure 3:**
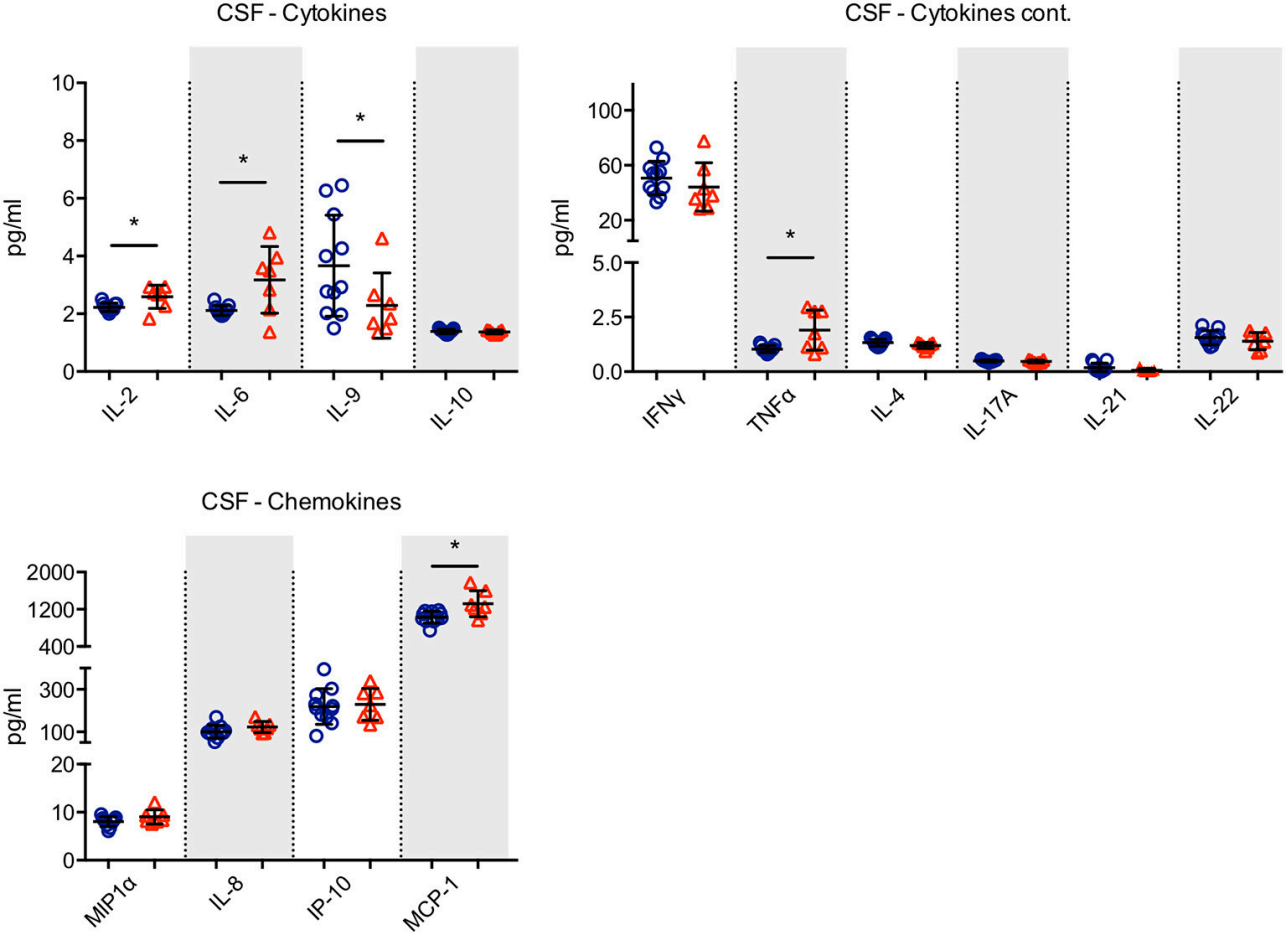
Cytokine and chemokine production in the cerebrospinal fluid. The concentrations of cytokines and chemokines were determined in the cerebrospinal fluid from healthy elderly controls (*n* = 11; blue circles) and PD patients (*n* = 7; red triangles) by a bead-based cytokine array. We found a significant increase of the pro-inflammatory cytokines IL-2, IL-6, and TNFα as well as of the pro-migratory chemokine MCP-1 in the CSF of PD patients, whereas anti-inflammatory IL-9 was decreased. For statistical analysis, the Mann-Whitney test was performed (*p < 0.05).

Using a bead-based cytokine array, we detected no significant differences in cytokine (IL-2, IL-4, IL-5, IL-6, IL-9, IL-10, IL-13, IL-17A, IL-17F, IL-21, IL-22, IFNy, TNFa) and chemokine (CCL11, CCL17, CCL20, CXCL1, CXCL5, CXCL9, CXCL11, IL-8, IP-10, MCP-1, MIP-1a, MIP-1b, RANTES) levels in the serum (data not shown) of PD. Interestingly, we found an increase of the pro-inflammatory cytokines IL-2, IL-6, and TNFα as well as of the pro-migratory chemokine MCP-1 (monocyte chemoattractant protein-1) in the CSF of PD patients, whereas anti-inflammatory IL-9 was decreased (Figure 3 A+B, only detected cytokines and chemokines shown).

## Discussion

In summary, phenotyping of CSF immune cells by multiparameter flow cytometry in patients with PD revealed a strong phenotypical shift of intrathecal monocytes and an increased percentage of activated T lymphocytes. In accordance, the levels of pro-inflammatory cytokines and MCP-1 were higher in the CSF of PD patients.

Evidence for a response of the innate immune system in the disease course of PD has been reported previously by neuropathological and functional brain imaging studies, genetics and CSF cytokine profiling assays (22). The hallmark response of innate immunity had been increased microglia activation and microglia recruitment to affected brain areas (13). Our results showing a strong shift from classical (CD14^+^CD16^−^) to non-classical (CD14^+^CD16^+^) monocytes within the intrathecal compartment represent a novel line of evidence for a response of innate immunity within the CNS. In contrast to a previous study that reported a significant increase in the proportion of PB classical monocytes in patients with PD (30), we found no significant differences in the proportion of peripheral monocyte subtypes. Monocytes originate from myeloid precursors in the bone marrow and are divided into two major subpopulations, the classical CD14^+^CD16^−^ and the non-classical CD14^+^CD16^+^ monocytes (31). Classical monocytes are highly plastic and, upon recruitment to inflamed tissue, modify their phenotype according to the requirements of the specific microenvironment. They can differentiate into macrophages and are involved in tissue maintenance, pathogen clearance and induction of adaptive immune responses. Non-classical monocytes are thought to patrol along blood vessels and to be involved in tissue homeostasis and local regeneration, however recent reports describe them as the primary inflammatory monocyte subtype with properties for antigen presentation (32, 33). Together, our data showing a strong increase in the proportion of CSF, but not PB, non-classical monocytes in patients with PD and previous evidence of increased classical monocytes in the PB of patients with PD suggest the occurrence of specific intrathecal monocyte activation. Thus, it is tempting to speculate that in PD classical monocytes are recruited from the periphery across the blood-liquor barrier to give rise to non-classical monocytes within the CSF.

Changes in the chemokine profile have been reported in PD patients in different stages of the disease (34). Interestingly, we found increased levels of MCP-1 (CCL-2) in the CSF of PD patients. The MCP-1 receptor CCR2 is expressed on monocytes and MCP-1/CCR2 signaling is involved in the regulation of migration and infiltration of monocytes into host tissues (35). Increased levels of MCP-1 had been previously described in both serum and CSF samples of PD patients, and higher levels of MCP-1 correlated with manifestation of cognitive impairment and depression (30, 36, 37). However, only classical monocytes express high levels of CCR2. Therefore, the shift in monocyte subsets might be related (1) to an enhanced evasion of classical monocytes into the brain tissue or (2) classical monocytes increasingly developing into non-classical monocytes. We found no evidence for a peripheral expansion of non-classical monocytes and their subsequent migration into the CNS since the specific monocyte subtype composition was confined to the CSF compartment. Monocytes invading the CNS might develop into macrophages within the brain parenchyma, thus strengthening the innate arm of the CNS immune system (30, 38).

In addition to the well-established response of innate immunity, more recent evidence has suggested a role for the adaptive immune system in PD. Genome-wide association studies have established an association of PD with alleles of the major histocompatibility complex (MHC) (23). Previous PB immune cell phenotyping had demonstrated increased LRRK2 levels in peripheral lymphocytes, which are involved in the regulation of T cell activation and division (39). Neuropathological studies have reported T cell infiltration into the substantia nigra (13). Interestingly, quantitative analysis demonstrated a substantial accumulation of CD8^+^ T lymphocytes and to a lesser degree of CD4^+^ T lymphocytes within the substantia nigra, although animal models suggested a more enigmatic functional role of the latter in the disease process (10). Our data support the neuropathological post-mortem results and provide the first direct *in vivo* evidence in man showing increased invasion of T lymphocytes into the intrathecal compartment and their functional activation. Recently, an elegant series of immunological studies led to the current concept of the contribution of T lymphocytes to the pathogenesis of PD: degenerating dopaminergic neurons present modified α-synuclein-derived peptides via MHC class I molecules on their surface and release them to the extracellular space, where they activate microglia, and drain into the periphery outside the CNS. In lymph nodes, they activate antigen-presenting cells, which present the α-synuclein fragments via MHC II surface molecules leading to T lymphocyte activation. The latter infiltrate the CNS and accumulate at sites of inflammation, where they are re-stimulated by α-synuclein-presenting neurons and microglia. This may result in exacerbated inflammation, oxidative stress, and neuronal injury (7, 10, 40, 41). Our data contribute to these findings by providing direct *in vivo* evidence for activated T lymphocytes in the intrathecal compartment.

In accordance to previous studies, we found increased levels of the pro-inflammatory cytokines IL-2, IL-6, and TNFα (12, 20). However, we were not able to detect a cytokine pattern indicating the enrichment of a specific T-helper (T_H_) subtype. Interestingly, IL-9 was decreased in PD patients, a pleiotropic cytokine with supposed regulatory effects in CNS autoimmunity (42). Low patient numbers and the assays' detection limits might affect our results or explain differences between them and previous studies. Concomitant autoimmune diseases, anti-inflammatory co-medication, or evidence of an acute inflammatory process at the time of CSF withdrawal were exclusion criteria for patients in the present study. However, we cannot exclude the possibility of unknown comorbidities that may affect results in the presented patient cohort.

In conclusion, our results demonstrate a shift of monocyte subsets and activation of T lymphocytes in the CSF of PD patients. Although it remains unclear whether such alterations play a primary or secondary role in neurodegeneration, our results provide a new tier of evidence for activation of both innate and adaptive immune responses in the disease course of PD. Moreover, dysregulated immune cells may represent interesting molecular targets accessible as biomarkers for the identification of disease-associated neuroinflammatory processes and amenable to therapeutic intervention. Such intervention could have positive clinical effects and potentially modify the disease course. Future studies are necessary to corroborate our findings. Correlation analysis based on larger cohorts will be necessary to link CSF immune cell responses to PD disease stages and other clinical and paraclinical parameters. Additional cellular surface markers and translational research will be required to unveil the cascade of events leading to altered CSF monocyte and lymphocyte phenotypes and define their origins and exact cellular identities.

## Author Contributions

JBS conceived, organized and executed the research project and reviewed the manuscript. MP conceived the research project, performed data analysis and wrote the first draft of the manuscript. GM helped to conceive the study and reviewed the manuscript. CCG performed data analysis and reviewed the manuscript. HW helped to conceive the study and reviewed the manuscript. SGM helped to conceive the study and reviewed the manuscript. TR conceived the research project, performed data analysis and reviewed the manuscript. TW conceived and supervised the research project and reviewed the manuscript.

### Conflict of Interest Statement

GM has received speaker honoraria and compensation for serving on Scientific Advisory Boards for LFB Pharma and Alexion Pharma. GM received research support from the Deutsche Forschungsgemeinschaft (DFG, grant number ME4050/4-1), from the Gemeinnützige Hertie Stiftung, from the Innovative Medical Research (IMF) program of the Westfälische Wilhelms-University Münster, and from the Ministerium für Innovation, Wissenschaft und Forschung (MIWF) des Landes Nordrhein-Westfalen. CCG received speaker honoraria and travel expenses for attending meetings from Bayer Health Care, Genzyme, and Novartis Pharma GmbH. HW received compensation for serving on Scientific Advisory Boards/Steering Committees, for Bayer Healthcare, Biogen Idec, Sanofi—Genzyme, Merck Serono, and Novartis. He has received speaker honoraria and travel support from Bayer Vital GmbH, Bayer Schering AG, Biogen, CSL Behring, EMD Serono, Fresenius Medical Care, Genzyme, Merck Serono, Omniamed, Novartis, and Sanofi Aventis. He has received compensation as a consultant from Biogen Idec, Merck Serono, Novartis, Roche, and Sanofi-Genzyme. HW also received research support from Bayer Healthcare, Bayer Vital, Biogen Idec, Merck Serono, Novartis, Sanofi—Genzyme, Sanofi US, and TEVA Pharma as well as the German Ministry for Education and Research (BMBF), Deutsche Forschungsgesellschaft (DFG), Else Kröner Fresenius Foundation, Fresenius Foundation, Hertie Foundation, Merck Serono, Novartis, NRW Ministry of Education and Research, Interdisciplinary Center for Clinical Studies (IZKF) Muenster, RE Children's Foundation. SGM received honoraria for lecturing, travel expenses for attending meetings, and financial research support from Almirall, Bayer Health Care, Biogen, Diamed, Genzyme, Merck Serono, Novartis, Novo Nordisk, ONO Pharma, Roche, Sanofi-Aventis, and Teva. TR received travel reimbursements from Merck Serono and financial research support from Sanofi Genzyme and Novartis and honoraria for lecturing from Sanofi Genzyme, Roche, Biogen, Merck, and Teva. TW has received lecture fees from UCB, Zambon, Bial, Licher, Teva, Bayer, and Abbvie, and worked as a consultant for Archimedes, UCB, and Abbvie. The remaining authors declare that the research was conducted in the absence of any commercial or financial relationships that could be construed as a potential conflict of interest.
