# Supplementary figures and images for: Immune Cell Activation in the Cerebrospinal Fluid of Patients With Parkinson's Disease
Source: Front Neurol. 2018 Dec 18;9:1081. doi: 10.3389/fneur.2018.01081 (PMC6305582; doi:10.3389/fneur.2018.01081)

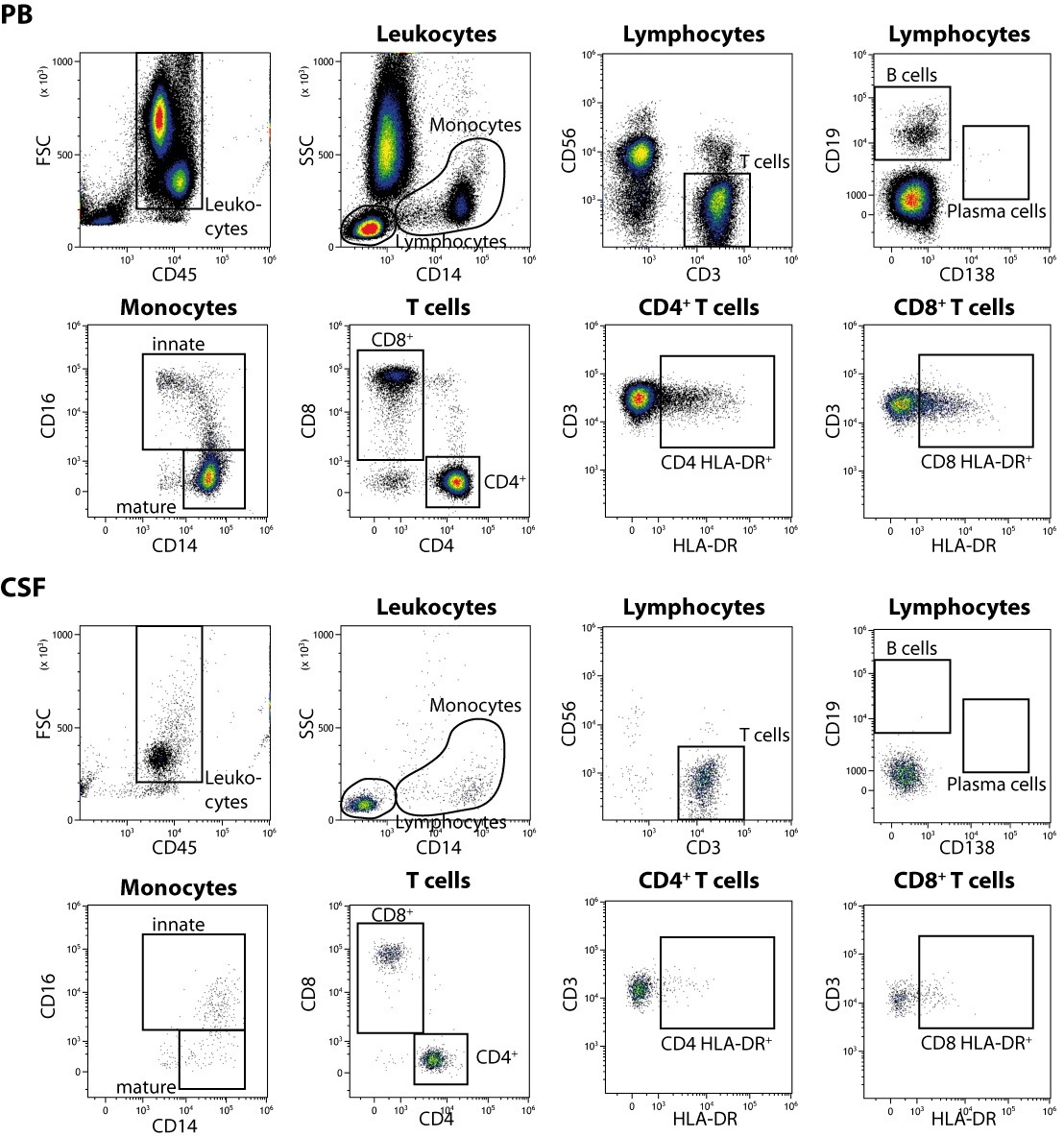

Supplement: Supplementary file 1 [file Image_1.TIFF]
